# Integrating ‘undetectable equals untransmittable’ into HIV counselling in South Africa: the development of locally acceptable communication tools using intervention mapping

**DOI:** 10.1186/s12889-024-18471-4

**Published:** 2024-04-15

**Authors:** Tembeka Sineke, Jacob Bor, Rachel King, Idah Mokhele, Mandisa Dukashe, Dorah Bokaba, Robert Inglis, Sharon Kgowedi, Bruce Richman, Cameron Kinker, John Blandford, Robert A.C. Ruiter, Dorina Onoya

**Affiliations:** 1 Health Economics and Epidemiology Research Office, School of Public Health, Faculty of Health Sciences, University of the Witwatersrand, Johannesburg, South Africa; 2 Department of Global Health, Boston University School of Public Health, Boston, MA USA; 3 Institute for Global Health Sciences, University of California, San Francisco, USA; 4 South African National AIDS Council (SANAC), Johannesburg, South Africa; 5 Tshwane Department of Health, City of Tshwane, South Africa, City of Tshwane, South Africa; 6 Jive Media Africa, Durban, South Africa; 7 Prevention Access Campaign, United States of America, New York City, USA; 8 Centers for Disease Control and Prevention, Johannesburg, South Africa; 9 Faculty of Psychology and Neuroscience, Maastricht University, Maastricht, the Netherlands

**Keywords:** Intervention mapping, U = U, Treatment-as-prevention, Intervention, South Africa

## Abstract

**Background:**

The global campaign for “Undetectable equals Untransmittable” (U = U) seeks to spread awareness of HIV treatment as prevention, aiming to enhance psychological well-being and diminish stigma. Despite its potential benefits, U = U faces challenges in Sub-Saharan Africa, with low awareness and hesitancy to endorse it. We sought to develop a U = U communications intervention to support HIV counselling in primary healthcare settings in South Africa.

**Methods:**

We used Intervention Mapping (IM), a theory-based framework to develop the “Undetectable and You” intervention for the South African context. The six steps of the IM protocol were systematically applied to develop the intervention including a needs assessment consisting of a systematic review and qualitative research including focus group discussions (FGD) and key informant (KI) interviews. Program objectives and target population were determined before designing the intervention components and implementation plan.

**Results:**

The needs assessment indicated low global U = U awareness, especially in Africa, and scepticism about its effectiveness. Lay counsellors and clinic managers stressed the need for a simple and standardized presentation of U = U addressing both patients’ needs for encouragement and modelling of U = U success but also clear guidance toward ART adherence behaviour. Findings from each step of the process informed successive steps. Our final intervention consisted of personal testimonials of PLHIV role models and their partners, organized as an App to deliver U = U information to patients in primary healthcare settings.

**Conclusions:**

We outline an intervention development strategy, currently in evaluation stage, utilizing IM with formative research and input from key U = U stakeholders and people living with HIV (PLHIV).

**Supplementary Information:**

The online version contains supplementary material available at 10.1186/s12889-024-18471-4.

## Introduction

Antiretroviral therapy (ART) significantly lowers the risk of HIV transmission to HIV-negative partners [1, 2], with zero risk of sexual transmission when the person living with HIV (PLHIV)’s viral load is undetectable [1–4]. While all 7.8 million PLHIV in South Africa are eligible for ART in 2022, 94% of people living with HIV knew their status, 76% of people living with HIV are on antiretroviral treatment (ART), while 92% of the estimated proportion of patients on ART were virally suppressed at 12 months [5–7]. The “Undetectable equals Untransmittable” (U = U) Campaign disseminates the science of HIV treatment as prevention (TasP) and has been endorsed by the U.S. National Institutes of Health (NIH) and by ministries of health in nearly 100 countries [8]. Sharing information on U = U could benefit the psychological well-being of PLHIV, reduce stigma, and motivate long-term ART adherence [9, 10].

Awareness of TasP and U = U is low in Sub-Saharan Africa where HIV prevalence is highest [11]. In South Africa (SA), HIV counselling has not historically emphasized the prevention benefits of ART [12]. There is hesitancy to endorse the U = U message for dissemination due to concerns such as lack of locally acceptable communication tools; lack of confidence in the science of U = U and the perception that U = U contradicts existing messaging on condoms [13]. At the same time, integrating U = U into HIV counselling could increase demand for ART, leading to greater ART uptake, adherence, and viral suppression. There is thus a critical need to develop U = U communications interventions that are scientifically accurate, theoretically-informed, locally acceptable, and contextually relevant to sub-Saharan African settings.

We sought to develop a U = U communications intervention to support HIV counselling in primary healthcare settings in South Africa. We used the Intervention Mapping (IM) [14] protocol to guide the development of an intervention that would be acceptable to PLHIV and health care providers, convey the science of U = U while addressing concerns, show how U = U can be integrated into the daily lives of PLHIV and their partners in SA, and model language for health care providers (HCPs) to counsel on U = U. IM constitutes a structured and iterative methodology for the development of interventions, adhering to six distinct and well-defined steps [14, 15]. It is used to create evidence-based interventions that are theoretically grounded, contextually relevant, and effectively tailored. This paper describes the intervention development process, theoretical framework, insights from formative data, and the key messages and design features of the intervention.

## Methods

### Study setting

The intervention was designed for dissemination to PLHIV receiving care at primary health clinics (PHCs) in South Africa. Formative research was carried out at three peri-urban PHCs in Johannesburg, South Africa, including PLHIV and providers of HIV care. Formative research was also conducted with members of an HIV civil society organization that seeks to educate people on U = U and with counselors providing care at other sites. The intervention was developed in collaboration with a local public health media and communication specialist, a local app software developer, and with feedback from stakeholders at local, provincial, and national government and the South African National AIDS Council (SANAC), including clinicians and PLHIV.

### Intervention mapping

IM (more from www.interventionmapping.com) is a systematic approach to multilevel intervention development and evaluation using grounded and participatory processes [14, 16]. IM includes six steps: (1) formative research to assess needs and (2) to identify intervention objectives, (3) selecting theory-based behaviour change methods and their practical application, (4) developing the intervention, and planning for (5) implementation and (6) evaluation.

### Intervention development working group (IDWG)

To ensure that the intervention was locally appropriate and contextually relevant, we constituted an Intervention Development Working Group (IDWG), a key feature of the IM protocol. The IDWG was comprised of key U = U and ART program stakeholders and experts, including the research team, PLHIV, and HCPs from the study sites (lay counsellors, nurses, their facility and district health managers), audio-visual communications content specialists, and representatives from civil society group of PLHIV. Throughout 2021, the IDWG convened on five occasions to systematically navigate the six steps of Intervention Mapping (IM). The group discussed the theoretical, contextual, and scientific underpinnings for the intervention [17], provided feedback on our literature review and qualitative data analysis in step 1. Subsequently, the IDWG defined intervention objectives and key messages in step 2, identified themes key approaches to deliver the material in step 3, and in step 4, selected participants for the videos, provided feedback on the scripts and videos as they were developed. In steps 5 and 6, the IDWG assisted in identifying forums to share the intervention with government and civil society in order to obtain feedback and plan towards broader implementation and evaluation.

### Conceptual framework

**Fig. 1:**
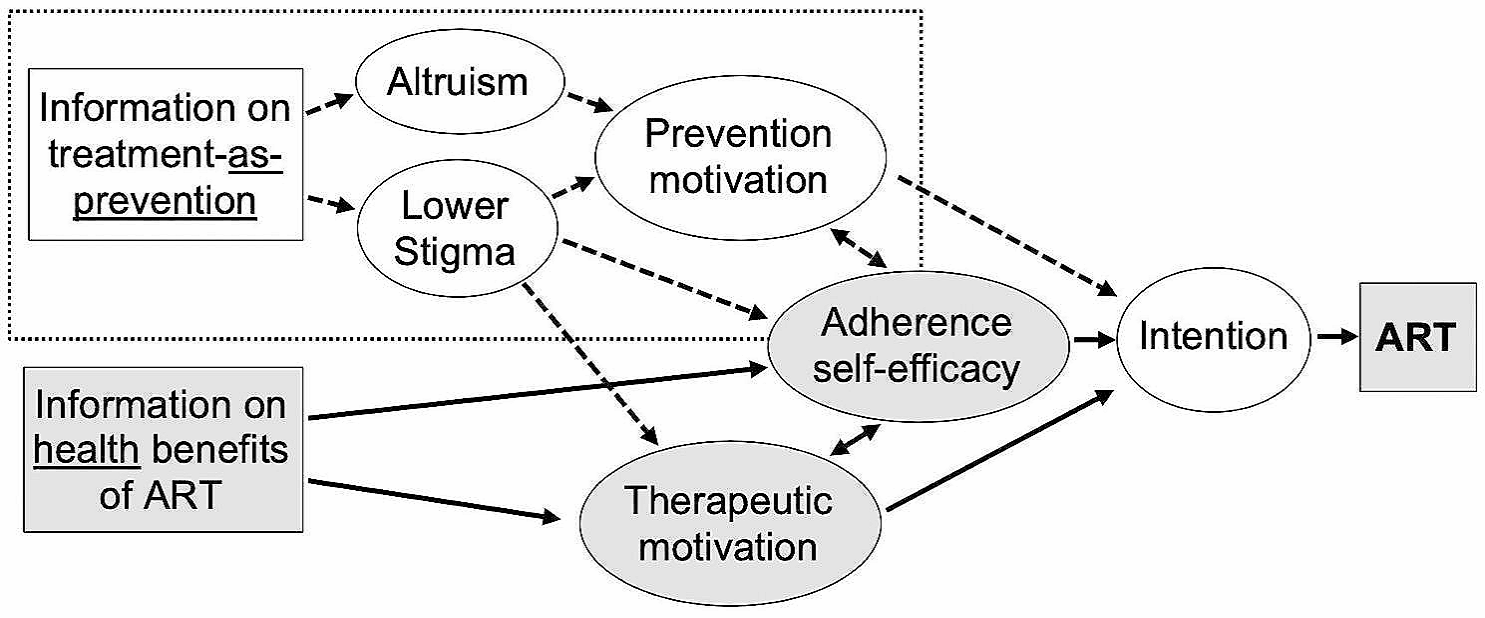
Conceptual model

Our conceptual model (Fig. 1) was based on the Information-Motivation-Behavioural Skills (IMB) model [18, 19] and the Theory of Planned Behaviour (TPB) [20]. These models focus on factors influencing individuals’ decisions to perform recommended actions. The IMB model encompasses three central constructs influencing behaviour change: decision-relevant information and knowledge regarding the target behaviour, individual motivation, and behavioural skills. In our work, we have adapted and expanded the IMB model to accommodate dual motivations—both therapeutic and preventive—for ART uptake and adherence. While existing counselling predominantly emphasizes therapeutic motivations for ART, our intervention goes beyond by incorporating U = U information to activate prevention motivations for ART. This means that in addition to the individual benefiting from ART for their own health, our intervention ensures that they also understand the role ART plays in preventing transmission to others when viral load is undetectable. It promotes a broader understanding of the role of ART in both individual health and HIV prevention. This holistic approach has the potential to improve outcomes for individuals living with HIV and contribute to reducing HIV transmission rates in communities. The videos feature testimonials by PLHIV who serve as “role models” for HIV status acceptance, therapeutic and prevention motivations, as well as behaviours including ART adherence, viral monitoring, and discussing HIV with a partner. This comprehensive approach aims to boost PLHIV’s confidence for successful ART adherence and to successfully guide them on how to attain specified goals to achieve sustained viral suppression.

### Steps 1 and 2: formative research to assess needs and define intervention objectives

Our formative research involved two phases: a systematic review of the literature and the collection and analysis of qualitative data from in-depth interviews and focus groups discussions (FGDs) with PLHIV and HCPs.

#### Literature review

We reviewed global literature on TasP and U = U knowledge and attitudes, including among patients, healthcare providers, and wider community. After screening 885 studies for eligibility, we included 72 in our review. Studies were coded according to prespecified fields, including year, location, study population, and whether the study reported on knowledge, acceptability, or behavioural impact. Because studies reported results in a variety of ways, quantitative and qualitative data were summarized in a narrative format, extracting key themes. A detailed description of this systematic review is published elsewhere [11].

#### Qualitative research

We assessed healthcare worker and PLHIV’s unmet needs for U = U messaging and attitudes towards U = U through FGDs and in-depth interviews (IDI) when FDGs were unfeasible. FGDs for HCPs focused on their U = U knowledge, attitudes, and existing processes for communicating VL results and discussing U = U with new and returning PLHIV. The FGDs also identified barriers and opportunities for U = U communication in primary healthcare. The FGDs and interviews with PLHIV gauged PLHIV’s knowledge about VL suppression and U = U, motivations for using HIV treatment for prevention, how HIV affected sexual relationships, and what information they had received from healthcare workers about U = U. IDI’s additionally focused on PLHIV’s journey with their HIV status. We asked participants to reflect on their HIV diagnosis, what their HIV status meant to them and how, if at all, being HIV-positive affected their lives [21]. Anonymised transcripts were analysed thematically. Each transcript was coded by two coders independently, with initial themes drawn from IDI and FGD guides. Major and cross-cutting themes were identified and refined over several analysis workshops. In cases where differences in codes could not be reconciled, a third member of the research team was assigned to help resolve the differences. In total, we conducted five FGDs with healthcare workers (*N* = 64) including nurses and counsellors from PHCs in the Gauteng and Free State Provinces of South Africa, three FGDs (*N* = 27) with PLHIV recruited by snowball sampling from civil society organizations, and 27 IDIs with recently diagnosed PLHIV in Johannesburg. Further details on methods and study participants are published elsewhere [12, 21].

**Table 1:** Overview of PLHIV participants

	IDIs (post-HIV diagnosis)	FGD1 (Peer support network)	FGD2 (U = U advocates)	FGD3 (Peer support network)
	*N* = 27 (col%)	*N* = 9 (col%)	*N* = 6 (col%)	*N* = 12 (col%)
**Age at enrolment**				
18–30	24 (88.9)	0	6 (100.0)	0
30+	3 (11.1)	9 (100.0)	0	12(100.0)
**Gender**				
Females	23 (85.1)	9 (100.0)	6 (100.0)	N/A
Males	4 (14.9)	N/A	N/A	12(100.0)
**Recruitment source**				
Civil society organisation	0	9 (100.0)	6 (100.0)	12(100.0)
Primary health facility	27 (100.0)	0	0	0

**Table 2:** Overview of HCP participants

Type of provider	Total	
Lay counsellor trainers/supervisors	28	
PHC nurses	12	
Project Coordinator	3	
Social auxiliary workers	6	
Enrolled nursing assistant	1	
Technical advisor: Psychosocial support	2	
Peer educators	2	
Community Systems Technical Officer (CSTO)	10	
**Total**	**64**	

Based on our formative research– and under the guidance of the IDWG– we revised the conceptual framework and identified in IM-step 2 key objectives for the intervention at the level of behaviour and its psychosocial determinants [18, 20]. More specifically, using the information gathered from the needs assessment, the personal factors (beliefs, evaluations) associated with increased adherence and retention on ART were identified. These psychosocial determinants such as attitude, perceived norm, self-efficacy and skills were linked to the different sub-behaviours that constitute successful ART adherence. In IM language, these sub-behaviours are formulated as performance objectives. The product of step two is then a matrix of change objectives that combines performance objectives with relevant psychosocial determinants (see Table 1 in supplementary materials). Change objectives are the most immediate targets of intervention that describe what needs to change in the identified determinants to achieve the specified performance objective. The logic of change then describes that by achieving the performance objectives through learning activities that target relevant determinants successful ART adherence is guaranteed.

### Steps 3 and 4: design key features of intervention and identify theoretical constructs to be targeted

We held multiple workshops with the IDWG to reflect on and interpret the formative data. On this basis, the IDWG (a) determined the type of intervention communications material to develop; (b) the voices who would be included; (c) how the material would be packaged for delivery; and (d) key messages to target related to the constructs in the theoretical model, i.e. information, motivation, behavioural skills modelled by each story; (e) how to manage potential threats to acceptability; (f) other stakeholders to be consulted.

### Steps 5 and 6: developing plans for implementation and evaluation

#### Preliminary evidence of local acceptability and contextual relevance

The IDWG was engaged to identify goals and opportunities for the pilot implementation and wider adoption of the intervention. We conducted discussions with facility managers and lay HIV counselors who would likely be responsible for administering the intervention to PLHIV. We presented the intervention at provincial and national HIV treatment literacy forums organized by SANAC. Through these forums, we elicited feedback from government officials, healthcare providers, and members of civil society groups. This collaborative process not only strengthened the intervention with input from diverse stakeholders, but also added credibility and legitimacy to the intervention, cultivated public trust and cooperation, and led to a formal collaboration with the National Department of Health. Importantly, these engagements with various stakeholders commenced early in the process, rather than solely during the implementation stage.

#### Evaluation, planning a randomized pilot

We are currently testing the intervention in a randomized controlled pilot trial at three primary healthcare clinics (PHCs) in order to assess the feasibility, acceptability, and preliminary impact of the intervention. Adherence to ART visits and viral suppression are being assessed through data extracted from clinical charts, providing insights into the impact of the intervention on health seeking behaviour. Outcomes will be reported in a separate paper.

## Results

Using IM, we created a user-friendly web and mobile application that features real individuals and healthcare worker videos, all aligned with foundational U = U constructs, behavioural theory, and local needs. Insights from FGDs and IDIs as well as our conceptual framework guided the selection of persons featured in the videos, framing of the “stories” presented, and key messages, as well as operational features of the intervention.

### Step 1: needs assessment

The systematic review highlighted low global U = U awareness, especially in Africa. Existing randomized trials showed that U = U communication could reduce stigma and increase HIV testing, but none to date had shown impact on ART adherence or viral suppression [11]. Our FGDs and KIs with PLHIV indicated limited U = U knowledge and scepticism about its effectiveness. Low viral load awareness underscored the need for U = U information sharing and VL status disclosure support. PLHIV identified significant hardships associated with their HIV status– anxiety about transmitting the virus to others, challenges with disclosure, fear of rejection by partners– that could be modified through dissemination of U = U [21].

**Fig. 2:**
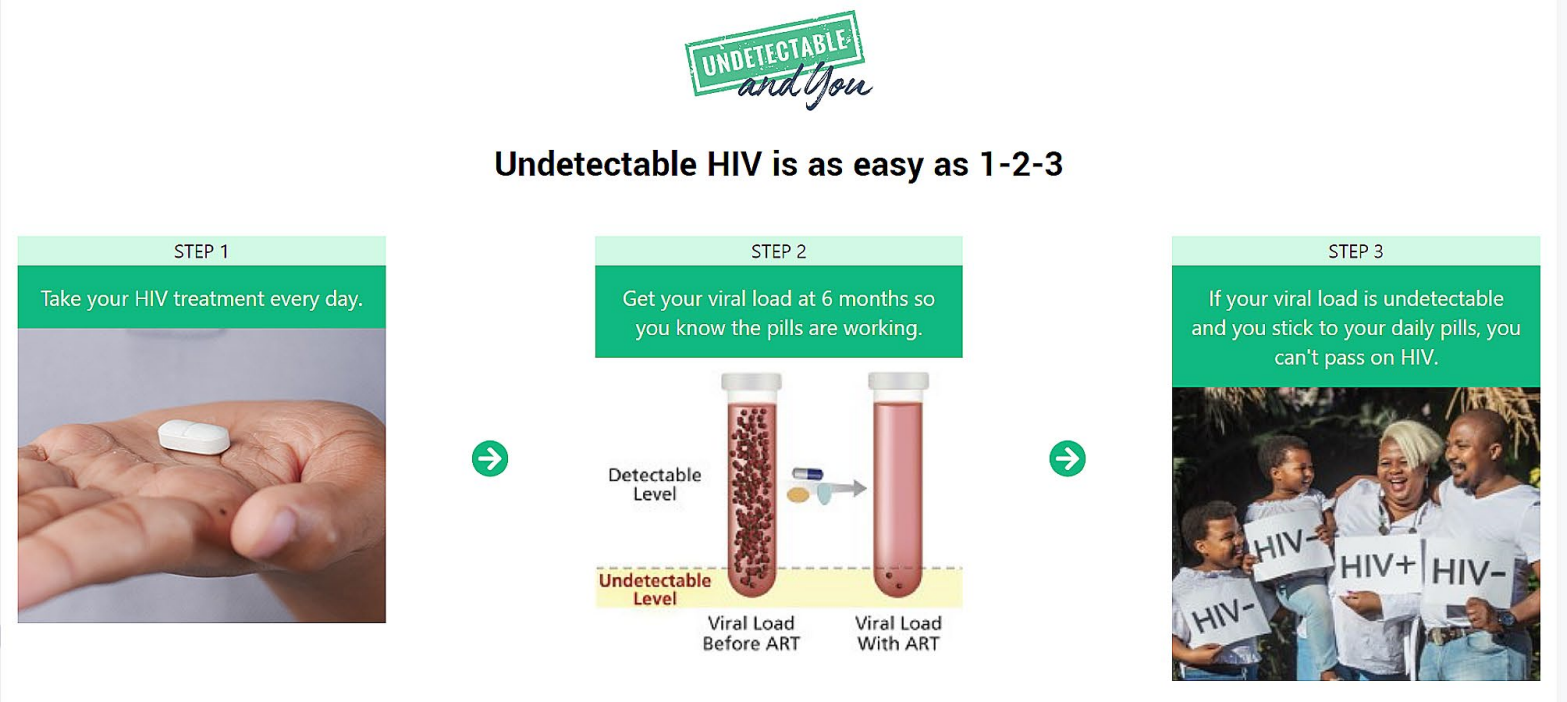
Screenshot from the “Undetectable and You” app

FGD with lay counsellors and clinic managers revealed varied U = U knowledge, stressing the need for simple and standardized presentation of U = U addressing both patients’ needs for encouragement and modelling of U = U success but also clear guidance toward ART adherence behaviour that support the efficacy of U = U in this context. HCP insisted on the need for information material that clarified the conditionality of U = U on viral suppression and was complementary to other prevention messages, especially related to condom use, to avert pregnancy, sexually transmitted infections, and HIV transmission in the event of an adherence lapse. We developed a simple, 3-step, linear process that emphasizes the conditionality of U = U on ART adherence and viral suppression (as identified through viral monitoring) (Fig. 2). Videos were designed both to provide information as well as to model accurate and compassionate U = U communication to build the skills of HIV counsellors.

### Step 2: intervention objectives

We developed a tablet-based application featuring a series of nine short video modules aimed at enhancing existing HIV counselling by emphasizing the prevention benefits of ART leading to viral suppression. These videos include testimonials from PLHIV regarding their U = U experiences, highlight motivations for adhering to ART, showcase diverse faces of the PLHIV community through relatable content and scenarios, and feature a HCW question and answer session modelling U = U communication. Accessible via a web application on phones and tablets, the app serves as a patient-facing tool during XXXcounselling sessions and facilitates peer-to-peer sharing of U = U information.

Based on our formative research, we identified the following intervention objectives:

1. *Increase Knowledge about U = U among PLHIV in primary health care clinics*.

- Emphasize the importance of viral suppression confirmed through a blood test for U = U validity.
- Highlight the critical role of daily ART adherence in achieving and maintaining viral suppression.
- Encourage the use of condoms in cases of uncertainties about viral suppression or for additional protection.

2. *Strengthen confidence in U = U and prevention motivations for ART adherence*.

- Present U = U as an antidote to self-stigma, affirming that individuals with suppressed viral loads cannot transmit HIV.
- Mitigate the fear of rejection by partners through reassurance that transmission risk is zero when viral load is undetectable.
- Instil hope and empowerment for PLHIV and their partners, dispelling uncertainties about their ability to have relationships, families planning, and lead fulfilling lives.

3. *Bolster Self-Efficacy in behaviours related to U = U*.

- Emphasize three simple steps (1-2-3) to confidently rely on U = U and the key role of viral monitoring.
- Build confidence in discussing U = U with others, empowering individuals to spread awareness and advocate for their well-being.

### Step 3: key features of intervention design

The IDWG determined key features of the intervention design. We decided that the intervention would feature videos with personal testimonials of PLHIV role models and their partners, organized as an App; that these role models would feature people with diversity in age, gender, sexuality, language/ethnicity, and appearance; and that these testimonials would address different motivations for U = U while targeting key theoretical constructs described below. It was determined that these testimonials would be interspersed with messages from a public health nurse who would deliver accurate information on the science of U = U, clarify and address potential misunderstandings of U = U, and model clear, compassionate, and non-judgmental health provider communication on U = U.

#### Clear and simple communication

Communicating U = U involves understanding HIV suppression through daily ART, linking transmission risk to viral load, and emphasizing that when the viral load is undetectable, transmission is impossible. The terms “undetectable” and “untransmittable” are complex and need clear explanations. To effectively convey this, it’s crucial to highlight the benefits of U = U and provide straightforward guidelines for people living with HIV. Clarity and simplicity are essential for broad accessibility and understanding.

#### Narrative story telling

During our formative research, we found that merely presenting the science of U = U isn’t sufficient; individuals need to grasp its benefits, find it relevant, and understand how to apply it in their lives. To address this, we employed narrative storytelling, featuring personal stories of different PLHIV. These testimonials, coupled with scientific information from a nurse, highlight the real-life impact and benefits of U = U. Our approach aligns with established theoretical models, emphasizing the importance of narratives and role models in promoting understanding and successful intervention.

#### Branding and reinforcement

To establish a personal connection, we use the slogan “Undetectable and You,” associating U = U with individuals’ everyday realities and fostering ownership. Titled “Undetectable and You,” our program personalizes U = U/TasP for PLHIV, fostering a strong association. The intervention includes a “test your knowledge” section with two scenarios: one for self-assessment and the other to reinforce app testimonials. Each scenario features multiple-choice questions. Scenario 1 involves selecting a partner with the lowest HIV transmission risk from four options, and Scenario 2 focuses on a discordant couple considering parenthood, followed by five related questions to reduce transmission risk. At the end of each videos, there is a three-step illustration that shows how people can achieve undetectable HIV and this includes: (1) “take treatment every day”, (2) “Get your viral load at 6 months so you know the pills are working.”, and (3) “If your viral load is undetectable and you stick to your daily pills, you can’t pass on HIV”.

#### Testimonials

The intervention program incorporates testimonials, role models which are presented in local languages and unfiltered real-life stories to inspire and empower participants. It draws strength from personal stories, offers guidance through role models, respects linguistic diversity, and promotes inclusivity through diverse hero representation. The videos also include a health care worker voice which delivers accurate science that aligns with NDoH guidelines and also models the U = U messaging for other health care workers.

#### Delivery mechanism

We use an App as a delivery mechanism for U = U information as this provide wide accessibility, reach and tracking capabilities. The App is administered during HIV counseling sessions and is available for patients to access on personal electronic devices such as smartphones and tablets outside the counselling session. These collectively make it a powerful tool for educating and reassuring individuals living with HIV about the significance of maintaining an undetectable viral load as a means to prevent transmission, ultimately enhancing public health efforts.

#### HIV diagnostic and adherence counselling

Additionally, the intervention focuses on PLHIV during two pivotal stages: HIV diagnostic counselling and adherence counselling. The central goal at these junctures is to emphasize the importance of achieving and sustaining viral suppression. In the initial phase, the intervention sensitively supports the HIV diagnosis counselling while providing information and promoting ART initiation and consistent ongoing adherence. This phase is crucial in helping PLHIV comprehend that viral suppression is achievable with proper treatment, promoting their well-being and reducing transmission risk. During ART adherence counselling, the intervention continues to prioritize viral suppression by addressing adherence challenges and concerns. It aims to empower individuals with the knowledge and resources necessary to maintain an undetectable viral load, reinforcing the U = U message.

### Step 4. intervention production

The intervention was designed to target key theoretical constructs identified in our conceptual model:

Our approach involved the thoughtful adaptation of theory-informed methods, moulding them into practical applications that later evolved into integral components of the final intervention program. These theoretical underpinnings not only informed but also propelled the practical strategies elaborated in (Table 2 in supplementary materials). In our approach, we incorporated well-established techniques such as chunking, advance organizers, visualization, tailoring, and active learning. These strategies were effectively employed to convey the intricate concepts of U = U in a manner that was accessible and comprehensible to the intended audience, enhancing the program’s overall impact.

- *Information on the Prevention Benefits of ART*.

- Clearly and unambiguously convey that U = U.
- Use PLHIV testimonials to convey real-life experiences and highlight how people can use ART as HIV prevention.
- Utilize a video featuring a healthcare worker to clarify the scientific concept of U = U and emphasize conditions for its effectiveness.
- Strategically align the message with promoting condom usage, and emphasize the importance of other methods to prevent other STIs and unwanted pregnancies.
  b. * Motivations for ART Uptake and Adherence*.
- Shift from altruistic desires to avoid transmitting HIV to a broader range of motivations linked to treatment as prevention.
- Emphasize motivations such as the desire for respect, control over the virus and one’s life, and optimism for the future.
- Showcase diverse motivations through dedicated videos embedding pertinent key messages from individuals of various backgrounds.
  c. *Behavioural Skills for U = U as a Prevention Modality*.
- Emphasize key behavioural skills: ART adherence, viral load monitoring and literacy, and discussing U = U with others.
- Communicate viral suppression in terms of daily ART adherence and highlight the importance of consistent daily commitment.
- Include a simplified three-step illustration for ensuring viral suppression, incorporating daily ART adherence and viral load testing.
  d. *Building Confidence and Self-Efficacy*.
- Ensure selected video candidates and their narratives are relatable and address real-life challenges associated with living with HIV.
- Articulate the “How” behind the integration of U = U into the candidate’s resilience journey, fostering self-efficacy.
- Use an authoritative healthcare worker (nurse) voice to expound upon the implementation of U = U and the conditions essential for its successful adoption.
  e. *Improving U = U Counselling Skills of Healthcare Providers*:
- Utilize real-life testimonials and consistent, supportive language from a healthcare worker as communication models for lay counsellors.
- Reinforce confidence in U = U and counselling patients effectively on U = U.

This integrated approach aims to provide a comprehensive understanding of U = U, supporting individuals in navigating and championing this transformative concept.

#### App’s design

The intervention serves to disseminate scientifically sound information on U = U in a culturally sensitive and tailored fashion. This approach is carefully crafted to ensure its acceptability among a diverse patient population, accommodating individuals with varying levels of literacy and numeracy skills. Moreover, the App’s design has been consciously crafted to resonate with healthcare workers, and its messaging is thoughtfully aligned to effectively address the concerns and inquiries we have gathered regarding the dissemination of U = U information. The U = U heroes underwent a meticulous selection process, ensuring that their stories were thoughtfully curated. This selection was driven by the goal of highlighting diverse reasons why U = U might hold significance for a wide range of individuals.

**Fig. 3:**
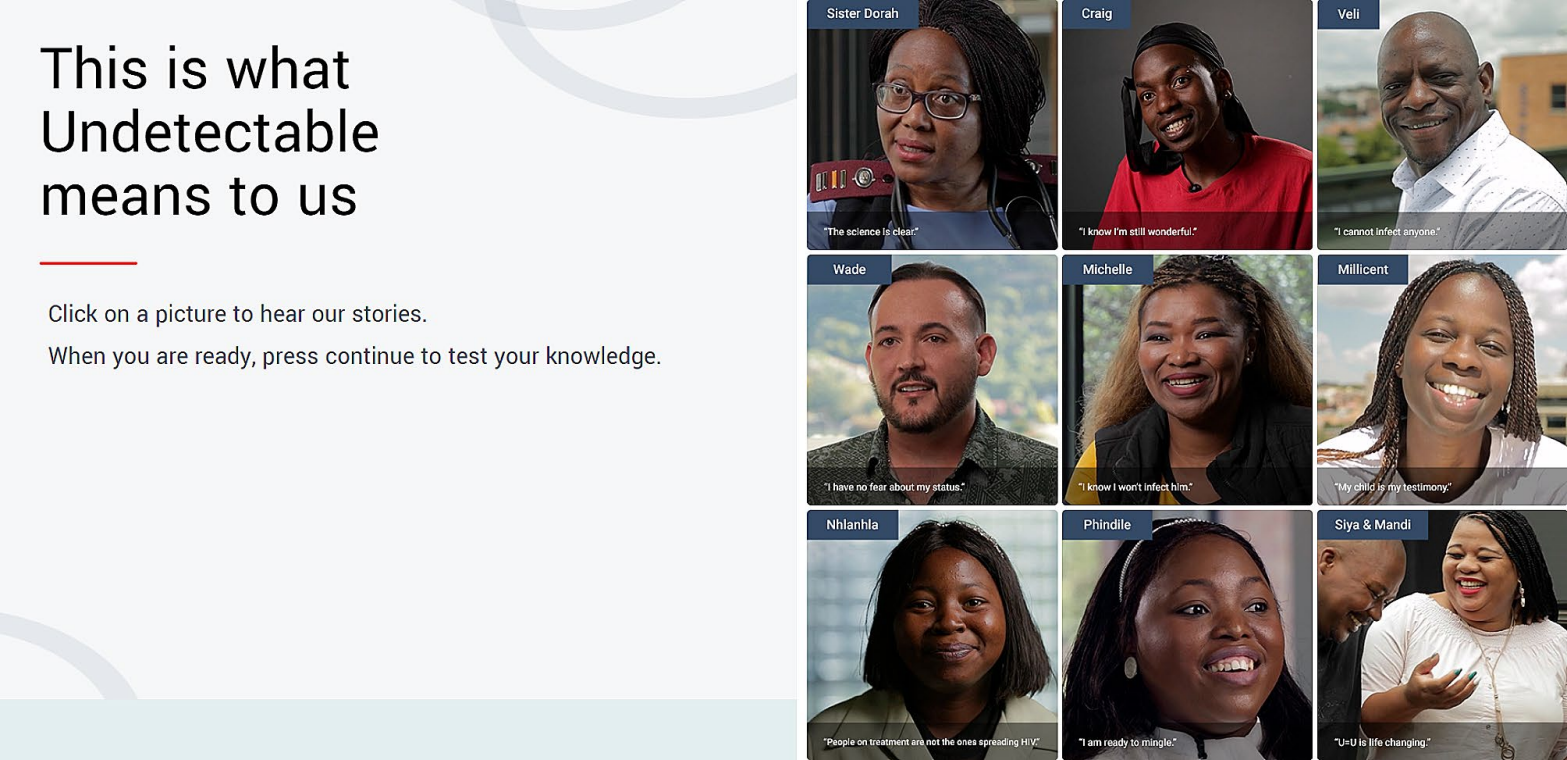
Screenshot from the “Undetectable and You” app

These stories were chosen to align with the various themes that emerged from our formative research, effectively capturing the multifaceted importance of U = U to different people as demonstrated below (Fig. 3). Veli struggled with self-stigma and reports that U = U made him “feel fully human again.” Millicent reports that having an HIV-negative child made her believe U = U was real: “my child is my testimony”. Nhlanhla sees U = U as a defence against external stigma: “People on ART like me are not the ones spreading HIV. Being virally suppressed means I love myself.” Phindile regains confidence in seeking a romantic partner now she knows she cannot pass it on if she takes her meds. Sister Dorah provides the scientific facts about U = U and counsels people that U = U does not protect against other STIs or pregnancy. She also models careful, accurate, and compassionate U = U communication for HIV counsellors.

### Steps 5&6: implementation and evaluation plan

The intervention has been implemented and is currently being tested in a randomized control trial across three primary health clinics and presented during SANAC meetings.

- *Pre-testing the intervention*.

In FGDs where we pretested the intervention, the content from the videos was perceived to be contextually relevant and to provide useful information. Content was perceived to be useful to gain a better understanding U = U and the meaning of viral suppression as demonstrated by the quotes below:

*“It will impact change in our lives, considering that we were not aware of some of the things and at least now there’s light, and we realise that you can actually live a normal life, have a family and live a normal life with someone who doesn’t have the virus. You can live a normal life without fear.”-****Participant#1, FGD***.

*“Firstly, adding to what was said by N#6, you have to stick to your treatment, secondly, it doesn’t mean by sticking to your treatment and having a low viral load count doesn’t mean you can’t get STI’s. you still can get affected because you’re still living like a normal human being. And the other thing she emphasised on in all her videos is self-control.…it doesn’t mean that because the virus is suppressed then it’s over. We need to be able to exercise self-control and not be reckless. She encourages us to always take care of ourselves.”****- Participant#2, FGD***.

*“I’m not worried about transmitting to someone else because I know that when I’m virally suppressed I can’t transmit, and I AM virally suppressed.”–****Male, civil society group***.

## Discussion

We developed a locally-acceptable, contextually appropriate U = U intervention rooted in theory and evidence for delivery in South Africa [14, 22]. The intervention, called “Undetectable and You”, was created to increase knowledge, reinforce motivations, and strengthen behavioral skills related to U = U. IM provided a valuable roadmap for intervention development, involving systematic collaboration with key experts and stakeholders to maximize potential for acceptability and impact.

We co-created the intervention with PLHIV and clinicians– the user populations for the intervention– as well as scientific experts from a range of disciplines. As members of our IDWG, these stakeholders were directly involved with the review of formative data, identification of intervention key messages and approach, and the development and refinement of the intervention videos.

Our systematic review of the existing literature, interviews and focus group discussions with HCWs and PLHIV, and consultations with the IDWG confirmed the rationale for the intervention– significant gaps in knowledge about U = U and a need for standardized, locally appropriate U = U communication tools. Our formative research helped us to refine our theoretical model, identifying key motivations for U = U and potential challenges to acceptability. Alongside our theoretical model, our formative data shaped the key messages and narrative structure of the videos as well as key design features of the intervention. Each of the theoretical constructs was targeted through a particular aspect of the intervention.

Our approach had several strengths. First, we used the IM process to guide intervention development. IM process has been successfully utilized in the development of interventions in various settings for different outcomes [12, 23–26] and also shown to be suitable framework for adherence self-management across conditions and treatments as it offered a transparent, systematic approach [27].

Second, our study was patient-centered [23, 26], underpinned by a behavioral model [24], and responsive to data collected from respondents that challenged our prior model. For example, our formative data highlighted locally specific motivations for U = U that were highly relevant to our population but had not been emphasized in the prior literature on U = U. Our intervention was collaboratively developed with PLHIV, who provided direct insight based on their own experiences. We also obtained input from implementers (HCPs). This inclusive approach is likely to enhance the intervention’s feasibility, acceptability, and effectiveness in promoting ART adherence.

Third, the testimonials feature a diverse set of people, with variation by age, gender, sexuality, race, and language. The language diversity in testimonials is essential for ensuring that the intervention is inclusive, culturally relevant, and accessible to a wide range of people. It helps in effective communication, outreach, and trust-building, ultimately contributing to the success and acceptance of the intervention in a linguistically diverse country like South Africa. Their testimonials are true, real-life stories, which are more relatable and contextually relevant and serve to contextualize the science of U = U in people’s lives.

Fourth, we packaged the videos into an easy-to-use web-based App. The App can be accessed on a personal device (phone, tablet) or a device at the clinic. The App makes it easy to collect de-identified data on utilization. It can also be updated with additional videos, features, or key messages as we collect data on how it is being used and its impact.

Fifth, the intervention is inexpensive to bring this to scale and integrate into existing programs and it can be implemented in primary care facilities by lay counsellors.

Our approach also had some limitations. Our intervention did not cover all 11 national languages but we included 3 that were widely spoken in our study setting and these include isiZulu, Sesotho, and English. This aligns with the goal of ensuring accessibility and comprehensibility of the intervention materials to the majority of patients visiting the PHC clinics in Gauteng. By focusing on the three most widely spoken languages in the study setting ensures the intervention’s practicality and effectiveness within the given context. We also conducted the study in one province out of the nine, but the province we included is very diverse one however with large gaps in viral suppression and a major need for adherence support interventions. Lastly, the App as currently developed requires access to Internet and a device but such access is widespread in health facilities and a growing share of the population has smart phones. Additionally, it is worth noting that the three PHCs included offer free internet access and the lay counsellors were also provided with data as part of the study.

A final limitation is that while acceptability has been very high, this paper does not include evaluation data and thus we cannot infer the effectiveness of the intervention on treatment adherence. Conducting a process evaluation will provide valuable insights regarding the acceptability and feasibility of intervention delivery in clinical practice, to further refine and improve the intervention.

## Conclusion

Our research contributes an intervention development approach and resulting intervention, currently under evaluation. IM provided a useful framework for applying formative research, existing literature and multidisciplinary input from IDWG for PLHIV. Disseminating information on TasP/U = U using locally-acceptable, effective interventions could reduce stigma increase ART uptake and adherence, leading viral suppression and preventing onward transmission of HIV.

### Electronic supplementary material

Below is the link to the electronic supplementary material.

Supplementary Material 1

Supplementary Material 2

## Data Availability

The datasets used and/or analysed during the current study are available from the corresponding author on reasonable request.

## Abbreviations

U=U: Undetectable equals Untransmittable
TasP: Treatment as Prevention
IM: Intervention Mapping
FGD: Focus Group Discussion
KI: Key Informant
IDWG: Intervention Development Working Group
PLHIV: People living with HIV
ART: Antiretroviral Treatment
NIH: National Institutes of Health
IMB: Information-Motivation-Behavioural Skills
TBP: Theory of Planned Behaviour
SANAC: South African National AIDS Council
PHC: Primary Health Clinic
NDoH: National Department of Health
STIs: Sexually transmitted infections
HCPs: Healthcare professionals
