# Supplementary material for: Integrating ‘undetectable equals untransmittable’ into HIV counselling in South Africa: the development of locally acceptable communication tools using intervention mapping
Source: BMC Public Health. 2024 Apr 15;24:1052. doi: 10.1186/s12889-024-18471-4 (PMC11020191; doi:10.1186/s12889-024-18471-4)
Supplement: Supplementary file 1 — Supplementary Material 1 [file 12889_2024_18471_MOESM1_ESM.docx]

**Supplementary Table 2. Theoretical methods for determinants translated in practical applications for intervention.**

| **Determinant** | **Theory-based method** | **Parameters for use** | **Practical application** |
| --- | --- | --- | --- |
| **Knowledge** | Chunking | Label assigned to aid with memory | Intervention App named “Undetectable and You” to associate TasP with the patients themselves and personalize it |
|  | Advance organizers | Schematic representation of content | Use of a three-step process of how to achieve a suppressed viral load and maintain suppression so that people can find it easy in their minds to remember how to attain it.  Use of B-OK bead bottles to explain viral suppression and the impact of ART on viral load. |
|  | Images | Images need to be familiar to a patient to help with an unfamiliar process | Familiar and relatable faces showing as thumbnails on the app as part of testimonials from ordinary people who talk about their personal experiences with using U=U |
|  | Active learning | Stimulation or activation by encouraging the learner to add to information to be remembered | Scenarios with multiple choice questions to test the knowledge after watching videos with testimonials.  The content in the videos promotes active learning. Participants choose the videos they want to watch and get to engage with the information from the videos. |
|  | Discussion | Make sure to listen to the patient to ensure that the correct schemas are activated | Counsellor speaks to a patient after administering the app to ensure that key information on U=U is well understood. The patient is given an opportunity to ask questions and discuss after being exposed to the App |
|  | Repeated exposure |  | Use of a total of 9 videos designed to convey U=U from different angles  Use of monthly text messages with reminder information about U=U within 6 months after enrollment |
| **Skills and self-efficacy** | Modelling | Attention, remembrance, skills, reinforcement; Credible source, method, and channel | Viewing the video explaining the science of U=U and testimonials from other PLHIV, the HCW video models how lay counselors should talk about U=U to patients.  For patients, the videos model what it is like to be a person who is virally suppressed. The patients can see themselves in others. |
|  | Reinforcement | Reinforcement that is individual and follows the desired behavior closely in time | Scenarios with multiple choice questions to test the knowledge after watching videos with testimonials. Answering the questions that come with scenarios helps reinforce the important concepts of U=U.  Use of repetition with a total of 9 videos designed to convey U=U from different angles and the HWC clip communicating the science appearing in all videos. |
|  | Feedback | Feedback that is individual, follows the desired behavior closely in time, and is specific | The use of an interactive app feedback that allows patients to give an answer to a scenario and positive encouragement for selecting the correct responses to scenarios/encouragement to keep trying to select the correct response if participants do not get the response right the first time |
| **Risk perception** | Scenario-based risk information | Plausible scenario with a cause and an outcome; imagery | Use of Scenarios detailing different risk profiles of hypothetical partners that patients are required to choose |
|  | Information about personal risk | Messages presented as individual, undeniable, on cumulative rather than for one occasion; messages presented with qualitative and quantitative examples | Use HCW video clearly explaining the risks of not adhering to ART and viremic state |
| **ART acceptability** | Information about the benefits of TasP/U=U |  | Use of authentic positive experiences with U=U to encourage patients to accept ART when offered. |
| **Motivation for ART** | Information about the benefits of TasP/U=U |  | Use of authentic positive experiences with U=U to elicit motivation to start and adhere to ART  Use of persuasive communication with testimonials that highlight the diversity and speak to the broad community of PLHIV |
| **Outcome expected** | Belief selection | Requires investigation of the current beliefs of the individual before choosing the belief on which to intervene | A measure of beliefs and attitudes about the use of TasP before intervention followed by exposure to the App and a measure of beliefs post-exposure to the intervention |
|  | Active processing of information | Individuals with high motivation and personally relevant, surprising, repeated, self-pacing, not distracting, easily understandable, and include direct instructions; messages that are not too discrepant and cause anticipation of interaction | Exposure to repeated science of U=U information seen in the different testimonials |
|  | Cultural similarity |  | Videos are in local languages and scenarios and created to sound relatable and realistic |
