# Supplementary material for: Integrating ‘undetectable equals untransmittable’ into HIV counselling in South Africa: the development of locally acceptable communication tools using intervention mapping
Source: BMC Public Health. 2024 Apr 15;24:1052. doi: 10.1186/s12889-024-18471-4 (PMC11020191; doi:10.1186/s12889-024-18471-4)
Supplement: Supplementary file 2 — Supplementary Material 2 [file 12889_2024_18471_MOESM2_ESM.docx]

**Supplementary Table 1- Matrix of change objectives for patients to start ART early and adhere to lifelong therapy**

Outcome objective - Patients start ART early and adhere to lifelong therapy

| **Performance Objectives** | **Knowledge** | **Attitude** | **Skills** | **Self-efficacy** | **Risk perception** | **Motivation for ART** | **Outcome expectation** |
| --- | --- | --- | --- | --- | --- | --- | --- |
| 1. Make a decision to get tested for HIV |  | Describe the importance of testing for HIV  Express a positive attitude towards testing for HIV |  | Express confidence to get tested for HIV | understand the risk of not getting tested | Express intention to get tested for HIV | Expect that testing for HIV will lead to a gateway to effective treatment |
| 1. Patients plan transport to get tested for HIV. | Knowledge of where to access the transport to the facility | Describe the importance of careful planning of transport | Demonstrate the ability to access transport |  |  |  | Expect travel to the clinic will result in testing for HIV |
| 1. Patients start treatment the moment it is offered by a lay counsellor | Knowledge of key benefits of starting ART early | Acknowledge the importance of an immediate start with treatment | Demonstrate an understanding of the benefits of starting treatment early | Feel confident in starting ART as offered after counselling | Recognize the risk of not starting ART early | Feel motivated to start treatment early as it is offered by counsellor | Expect that starting treatment early will prevent sickness and opportunistic infections  Describe the benefits of starting early  Describe prevention and therapeutic benefits of ART |
| 1. Patients keep ART with them at all times and know how to take it correctly | Knowledge about correct way of taking ART |  | Demonstrate ability to access ART at all times |  |  |  | Expect that if patient will remember to take ART if they have access to it at all times |
| 1. Patients take ART correctly and consistently | Knowledge of the importance of taking ART daily | Describe benefits of taking ART daily and what dosage of medication should be taken | Demonstrate understanding of reasons for taking ART daily as prescribed | Express confidence in using the ART consistently and correctly |  | Feel motivated to take ART correctly and consistently | Expect that ART is most effective by taking the right dosages, at the right time with the right interval |
| 1. Patients continue to take treatment effectively even in the case of side effects |  |  | Demonstrate the skills to manage the side effects  Describe strategies to cope with side effects | Express confidence in making a personalized coping plan to manage side effects and worries |  | Feel motivated to take ART despite side effects | Expect that side effects are manageable with own personalized coping plan and skills |
| 1. Patients adhere to lifelong treatment | Knowledge of adhering to lifelong treatment | Underscore the importance of lifelong adherence to ART | Demonstrate an understanding of the adverse effects of non-adherence to HIV prevention and the benefits of lifelong adherence for HIV prevention | Express confidence in the ability to adhere to ART | Recognize the risk of not adhering to lifelong ART |  | Expect adhering to lifelong treatment will improve the treatment outcomes  Expect that a routine helps to support optimal medication adherence  Describe the adverse outcomes resulting from interruption in medication intake |
| 1. Patients attend clinic visits as scheduled | Describe how attending the clinic visits will impact the overall treatment outcomes | Describe the viral load (VL) testing schedule  Describe the meaning and implications of viral load results | Demonstrate knowledge of the next clinic visit date | Express confidence in the ability to attend clinic visits as scheduled  Express confidence in making a personalized plan to remember scheduled clinic visits |  | Feel motivated to attend scheduled clinic visits | Expect that attending clinic visits as scheduled will improve treatment outcomes |
| 1. Patients adhere to viral load monitoring lab investigations |  |  | Demonstrate understanding of the link between non-adherence and viremic state | Feel confident in ability to adhere to monitoring labs |  | Feel motivated to adhere to monitoring labs | Expect monitoring viral loads will lead to better health outcomes |
| 1. Patients reduce the likelihood that they forget the treatment |  |  | Demonstrate understanding of daily treatment dosage and scheduling  List strategies to remember to take ART as prescribed |  |  |  | Expect adherence to ART will result to improved treatment outcomes  Describe the implications of non-adherence |
| 1. Patients cope with negative comments from the social environment |  | Describe ways to cope with discriminatory comments |  | Express confidence in applying coping mechanisms against HIV stigma | Identify coping skills that advantageous |  | Expect that implementing coping strategies will help with coping with stigma |
